# Supplementary material for: In Vitro Evaluation of the Antioxidant Activity and Chemopreventive Potential in Human Breast Cancer Cell Lines of the Standardized Extract Obtained from the Aerial Parts of Zigzag Clover (Trifolium medium L.)
Source: Pharmaceuticals (Basel). 2022 Jun 2;15(6):699. doi: 10.3390/ph15060699 (PMC9229722; doi:10.3390/ph15060699)
Supplement: Supplementary file 1 [file pharmaceuticals-15-00699-s001.zip › pharmaceuticals-1749945-supplementary.pdf]

pharmaceuticals

MDPI

Article

In Vitro Evaluation of the Antioxidant Activity and Chemopreventive Potential in Human Breast Cancer Cell Lines of the Standardized Extract Obtained from the Aerial Parts of Zigzag Clover (*Trifolium medium* L.)

Grażyna Zgórka [1,*], Magdalena Maciejewska-Turska [1], Anna Makuch-Kocka [2], Tomasz Plech [2]

[1] Department of Pharmacognosy with the Medicinal Plant Garden, Faculty of Pharmacy, Medical University of Lublin, 1 Chodźki Str., 20-093 Lublin, Poland; grazyna.zgorka@umlub.pl (G.Z.); magdalena.maciejewska@umlub.pl (M.M.T.)
[2] Department of Pharmacology, Chair of Pharmacology and Biology, Faculty of Health Sciences, Medical University of Lublin, 20-093 Lublin, Poland; anna.makuch@umlub.pl (A.M.K); tomasz.plech@umlub.pl (T.P.)
* Correspondence: grazyna.zgorka@umlub.pl.

Citation: Zgórka, G.; Maciejewska-Turska, M.; Makuch-Kocka, A.; Plech, T. In Vitro Evaluation of the Antioxidant Activity and Chemopreventive Potential in Human Breast Cancer Cell Lines of the Standardized Extract Obtained from the Aerial Parts of Zigzag Clover (*Trifolium medium* L.). *Pharmaceuticals* **2022**, *15*, 699. https://doi.org/10.3390/ph15060699

Academic Editor: Jong Heon Kim

Received: 15 May 2022
Accepted: 31 May 2022
Published: 2 June 2022

Publisher's Note: MDPI stays neutral with regard to jurisdictional claims in published maps and institutional affiliations.

Copyright: © 2022 by the authors. Submitted for possible open access publication under the terms and conditions of the Creative Commons Attribution (CC BY) license (https://creativecommons.org/licenses/by/4.0/).

Supplementary Materials

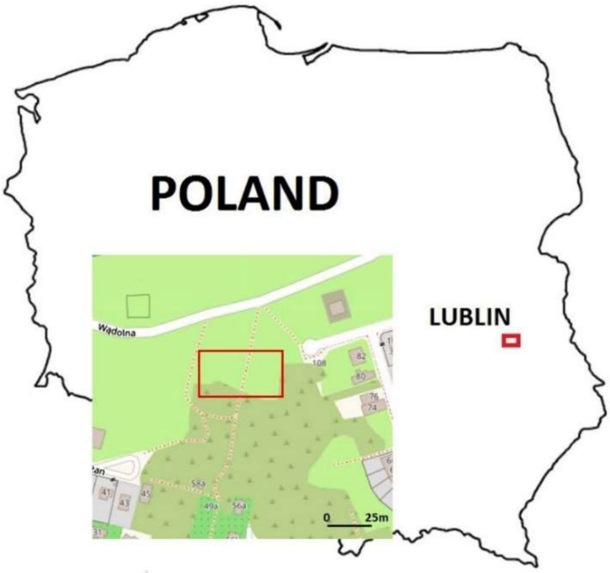

Figure S1. A collection site of wild zigzag clover (in red frame) against the contour map of Poland and a fragment of enlarged map of the city of Lublin

Table S1. Mean retention times (t_R), linearity parameters for calibration curves ($y = ax + b$), and *LOD* and *LOQ* values (λ = 260 nm) obtained for reference isoflavones using RP-LC/PDA method

**Isoflavone**	t_R (min)	*a*	*b*	R^2	*LOD* (ng/ml)	*LOQ* (ng/ml)
genistin	36.31	49167.58	-7.38	0.99994	86	287
ononin	43.57	36949.68	1.67	0.99996	123	410
sissotrin	54.64	51416.04	-3.05	0.99998	97	323
genistein	64.01	76505.19	2.38	0.99998	71	237
formononetin	68.19	53013.74	-4.72	0.99997	47	157
biochanin A	72.15	72150.31	-12.47	0.99997	28	93

Abbreviations: *a*- slope; *b*- intercept; R^2- regression coefficient; *LOD*– limit of detection; *LOQ*– limit of quantification

Table S2. Mean results (μg/g dry wt) obtained for TML isoflavones in intra- and inter-day precision assays using the RP-LC/PDA method

**Inter-day** (*n* = 9)	**Intra-day** (*n* = 3)							
**Isoflavones**	1st day		2nd day		3rd day			
	Mean	*RSD* (%)	Mean	*RSD* (%)	Mean	*RSD* (%)	Mean	*RSD* (%)
genistein	2001	1.2	2048	0.9	2037	1.4	2029	1.2
genistin	2247	1.3	2283	1.1	2299	1.2	2276	1.2
biochanin A	3525	1.9	3680	3.3	3603	1.1	3603	2.2
sissotrin	8360	1.2	8179	1.1	8278	0.9	8272	1.1
sissotrin malonate	20537	0.4	20618	0.2	20744	0.9	20633	0.5
formononetin	2963	2.7	2790	3.9	2989	4.6	2914	3.7
ononin	4409	2.3	4481	0.6	4337	1.9	4409	1.6
ononin malonate	7317	4.8	7012	3.4	7614	4.0	7314	4.1

Abbreviations: *RSD* – related standard deviation of the mean value (in %)
